# Quantitative material analysis using secondary electron energy spectromicroscopy

**DOI:** 10.1038/s41598-020-78973-0

**Published:** 2020-12-17

**Authors:** W. Han, M. Zheng, A. Banerjee, Y. Z. Luo, L. Shen, A. Khursheed

**Affiliations:** 1 Department of Electrical and Computer Engineering, National University of Singapore, 4 Engineering Drive 3, Singapore, 117583 Singapore; 2 Physics Department, Bidhan Chandra College, Kazi Nazrul University, Asansol, West Bengal 713303 India; 3 Department of Mechanical Engineering, National University of Singapore, 9 Engineering Drive 1, Singapore, 117575 Singapore

**Keywords:** Characterization and analytical techniques, Scanning electron microscopy, Electronic properties and materials, Imaging techniques

## Abstract

This paper demonstrates how secondary electron energy spectroscopy (SEES) performed inside a scanning electron microscope (SEM) can be used to map sample atomic number and acquire bulk valence band density of states (DOS) information at low primary beam voltages. The technique uses an electron energy analyser attachment to detect small changes in the shape of the scattered secondary electron (SE) spectrum and extract out fine structure features from it. Close agreement between experimental and theoretical bulk valance band DOS distributions was obtained for six different test samples, where the normalised root mean square deviation ranged from 2.7 to 6.7%. High accuracy levels of this kind do not appear to have been reported before. The results presented in this paper point towards SEES becoming a quantitative material analysis companion tool for low voltage scanning electron microscopy (LVSEM) and providing new applications for Scanning Auger Microscopy (SAM) instruments.

## Introduction

The development of low voltage scanning electron microscopes (LVSEMs) has transformed the subject of scanning electron microscopy over the last few decades. LVSEMs have many well-known advantages over conventional scanning electron microscopes (SEMs), such as higher signal yields, a smaller beam/specimen interaction volume, greater surface information, and the possibility of minimizing charging effects while inspecting non-conductive specimens^1–3^. These advantages, together with technological developments in electron sources, electron detectors and low aberration immersion/retarding field objective lens designs, have made it possible for SEMs to now operate with *nm*/sub-*nm* image resolution on a wide variety of different specimens^4–7^.

Historically, SEMs were operated with primary beam voltages in the 5–30 kV range, which not only limited their image resolution on bulk specimens to the micrometer range, but also prevented them from directly observing non-conductive specimens due to primary beam charging effects. This restriction has largely been overcome through the use of LVSEMs. LVSEMs are finding an increasing number of applications in subjects such as biology and chemistry, which involve observing organic samples directly. Precision imaging of this kind has uncovered new kinds of information, some recent examples of this being the imaging of multi-layer polymer surface micro-structures^8^, the ultrastructural surface topography of cell nuclear domains^9^, and the morphology of polymer-fullerene photovoltaic blends^10^.

Despite the many advantages of LVSEM over conventional SEMs, it is still primarily an imaging method, and does not naturally provide any material or chemical information about the sample. In conventional SEMs, the energy dispersive X-ray spectroscopy (EDX) technique is widely used to provide quantitative material analysis information. EDX functions by capturing X-ray spectral peaks emitted from the sample when it is bombarded by the instrument’s primary electron beam^11^. The technique usually operates with primary voltages of 6 kV and above. For primary beam voltages lower than 6 kV, the technique does not perform well due to the problem of the primary electron beam not having sufficient energy to create the necessary atomic transitions that emit X-rays^12^. Although some EDX detectors have been designed to operate at lower primary beam voltages, the range of specimens that they can observe is much more restricted^13–15^. This means that at present, there is no companion quantitative material science technique that can be used together with LVSEM, in a manner similar to the way EDX is presently used in conventional SEM.

The scattered low energy electrons (< 50 eV) generated inside a SEM by the primary beam striking the sample, known as secondary electrons (SEs), are usually used for the purpose of topographical imaging, while its higher energy (> 50 eV) back scattered electrons (BSEs) are commonly used for qualitative material contrast imaging^16^. Although material contrast information is present in the SE image, there is no clear identifiable relationship between specimen atomic number and SE yield^17,18^. The research work presented here involves placing an electron energy analyser attachment inside the specimen chamber of a conventional SEM in order to capture the lower part of the SE energy spectrum (0–20 eV) while operating in LVSEM conditions. Experimental results obtained from it demonstrate how it can be used to extract quantitative material analysis from the SE energy spectrum. In the past, electron energy spectral experiments were carried out in customised specialised Ultra High Vacuum (UHV) chambers, and they were able to demonstrate how SE energy spectroscopy (SEES) and angle resolved secondary electron energy spectroscopy (ARSEES) could capture electronic band structure information about a sample’s occupied and unoccupied density of states distributions^19–24^. Material science/chemical information of this kind today is normally acquired by techniques such as photoemission spectroscopy (PES) and angle-resolved photoemission spectroscopy (ARPES), and is not usually associated with the SEM^25^.

There have as yet, been very few electron energy analyser attachments specifically designed for SEMs. The specimen in LVSEMs is usually integrated into the objective lens assembly, and the space directly above it is not usually accessible. In conventional SEMs, the distance from the specimen to the objective lens lower pole-piece, known as the working distance, is usually made as small as possible (below say 20 mm) in order to maximise image spatial resolution. There is therefore, very little space into which any energy analyser attachment can be placed within current SEM designs. Due to these technical limitations, only a small number of electron energy analyser attachments have proved capable of carrying out secondary electron energy spectroscopy (SEES) in the SEM^26–29^. It should be noted that the ad-hoc attempts of using in-built LVSEM SE detection systems to energy filter the SE detector signal have succeeded in providing image enhancement, but have not proved precise enough to carry out SE energy spectroscopy^30–36^. This is because their output signals are influenced by tertiary electrons generated by internal electrode scattering and they detect SEs that have a complex mix of different emission angles and energies. Their spectral signals appear jagged, distorted and truncated when compared to the SE energy spectral signals obtained by dedicated UHV SE spectral systems^37–42^. The band-pass energy analyser designs commonly used in surface science spectral systems focus electrons having different emission angles on to a narrow slit lying on an exit energy dispersion plane, so that the influence of emission angle and spurious tertiary electron generation are suppressed^43,44^.

The work reported here utilises a small wide-angle electric toroidal energy analyser SEM attachment design^29^, which has formerly been used to track small changes in the SE energy spectral signal shape for applications such as quantifying dopant concentration^45,46^ and mapping interface/surface charge distributions^47,48^. It is used here to perform quantitative material analysis in a variety of different ways. Experimental results demonstrate how the SE spectral signal peak height and standard deviation can be used to estimate a metal sample’s elemental atomic number and distinguish between metal regions having close atomic numbers. This is the first time a simple analytical relationship between sample atomic number and secondary electron emission has been obtained. Most other attempts at finding such a relationship have so far been directed towards monitoring overall SE yield using hemispherical retarding field detectors in a UHV environment, and were not able to find any such simple relationship^17,18^. Near 100% quantitative material contrast between Pt and Au regions on a test sample was obtained by tracking changes in the SE energy spectral signal’s shape at a primary beam voltage of 1 kV, under conditions where no visible material contrast was observed in the conventional SEM detector image or EDX attachment map.

Experimental results presented here also show how fine spectral features in the lower energy part of the SE energy spectrum (0 to 10 eV) can be extracted and used to capture a sample’s valence band bulk density of states (DOS) distribution. This is the first time chemical information of this kind has been obtained in a SEM. This technique of finding SEM SE energy spectral DOS distribution functions by subtracting two different low primary beam voltage SE energy spectral signals from one another (the 1 kV one from the 0.5 kV one), and differentiating the residual spectrum with respect to SE energy. This procedure was found to successively suppress the influence of SE cascade interactions, leaving an output energy spectral signal that is able to directly represent the sample’s bulk valence band DOS distribution. A close match between this experimentally derived SE spectral DOS signal and theoretical valence band DOS data generated by Density Functional Theory (DFT) was obtained for six different test samples, W, Cu, Au, Pt, Al and Si. The normalised root mean square deviation (NRMSD) between experimental and theoretical DOS distributions for all these test samples ranged from 2.7 to 6.7%. Such high levels of agreement between experimental and theoretical bulk valence band DOS data do not appear to have been reported before.

The SEM SEES technique as presented here has obvious similarities to PES, which also provides DOS distribution information from the capture of scattered low energy electron spectral data, but there are some notable differences. The SEM specimen chamber operates in HV conditions, allowing it to use a wide range of different samples, and as long as the sample is pre-cleaned with acid, the experimental results presented here demonstrate that it can provide reliable accurate bulk information about a sample’s occupied energy states in the presence of surface contamination. Also, SEES inside the SEM naturally combines spectroscopy with nanoscale imaging. In contrast, PES requires UHV conditions and in-situ cleaning in order to minimise sample surface contamination. This is because its information comes mainly from surface states, and not from specimen bulk states^49^. In addition, PES is essentially a spectral electron energy method, and does not usually have imaging acquisition capability. More elaborate techniques at synchrotron facilities have been devised to achieve spatially resolved PES/ARPES^50^. Another advantage of the present SEM SEES technique over PES is that it captures valence band DOS distribution directly, without the presence of a residual SE cascade background signal. DOS spectral information in the PES output signal is usually embedded within a broader background cascade SE signal. Both this and its inherent surface sensitivity to contamination limit the precision to which PES spectra can be matched to theoretical DOS data.

In principle, the same method presented here for SEMs can also be used by Scanning Auger Microscopy (SAMs) instruments. Apart from their inherent UHV mode of operation, they, like the SEM, involve scanning a primary electron beam over a specimen’s surface for the purpose of acquiring an image, but in addition, have an in-built energy analyser for the purpose of acquiring scattered electron energy spectral information. However, they are not usually optimised to capture scattered electrons in the lower SE range (0–20 eV). A common problem preventing such systems being applied to capture the SE energy spectrum is that they require sample biasing for Auger electron spectroscopy (AES) calibration, which in turn, distorts the SE energy spectrum^51^. Special sample holders have been proposed in order to screen and reduce this effect^52,53^, but even where such precautions have been taken, experimental SE energy spectra obtained by SAMs for the same primary beam voltage (0.5 kV) and test metal samples used here (W, Cu, Au and Pt), have not shown the presence of valence band fine spectral features^54^. In the subject of surface science, extraction of valence band information from the lower part of the SE energy spectrum has so far only been successively obtained by the construction of specialised customised SEES UHV chambers which did not have any imaging capability^19,20^. Apart from becoming a material analysis tool for LVSEM, the present SEM SEES technique may therefore also point the way towards a new line of development for instruments such as SAMs.

It should be noted that although the research work reported here was carried out in a conventional SEM, several electron energy analyser designs have been proposed for in-lens SEMs, and some of them have obtained proof-of-concept experimental SE energy spectral signals^55–58^. The proposition of SE energy spectroscopy becoming a quantitative material analysis for LVSEM is therefore equally relevant to the state-of-the-art SEM column designs.

## Results and discussion

### Quantitative material contrast using SE energy spectral signal shape changes

**Figure 1:**
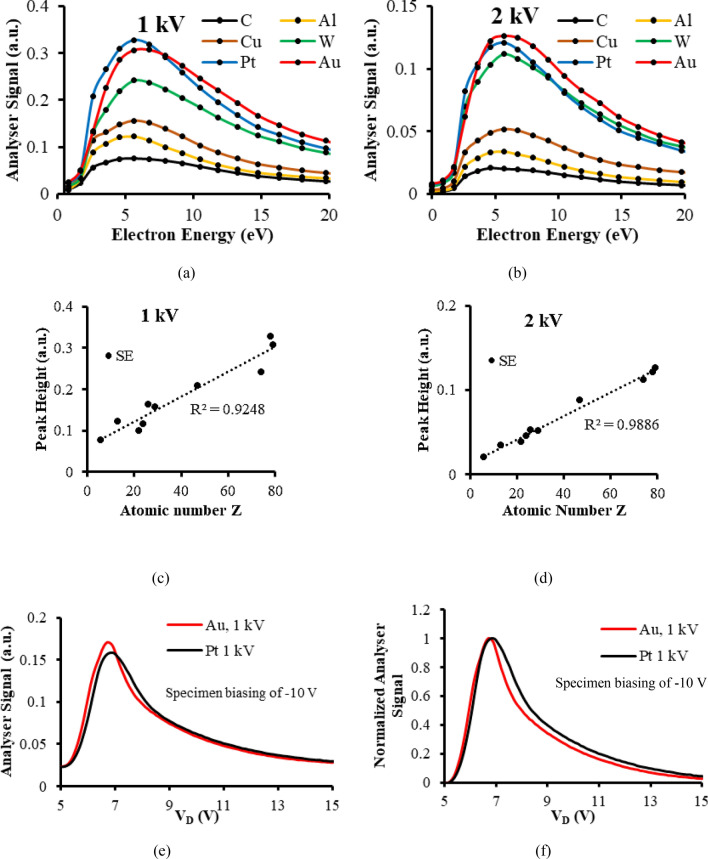
Quantitative material contrast extracted from energy analyser spectral signals (**a**) Experimental energy analyser spectra at a primary beam voltage of 1 kV for 6 different metals with 0 V specimen bias (**b**) Experimental energy analyser spectra at a primary beam voltage of 2 kV for 6 different metals with 0 V specimen bias (**c**) Spectral peak height variation with atomic number at a primary beam voltage of 1 kV (**d**) Spectral peak height variation with atomic number at a primary beam voltage of 2 kV (**e**) Experimental energy analyser spectra at a primary beam voltage of 1 kV for Pt and Au metal foil specimens with a − 10 V specimen bias (**f**) The normalised experimental energy analyser spectra shown in (**e**).

Figure 1a,b present experimental SE energy spectra taken at the primary beam voltages of 1 and 2 kV for 6 different test specimens. These analyser spectral signals *S(E)*, do not represent the emission SE energy distribution leaving the specimen surface, *N(E)*, but are proportional to *E* × *N(E)*, as explained in Supplementary Sect. 1. Material contrast information is present in terms of spectral signal height and shape changes. The 1 kV spectra have higher peak values than the 2 kV spectra, and appear to be less smooth for lower energies (< 5 eV). The spurious background signal at 0 eV generated from high energy multiple scattering events within the energy analyser is retained in both cases. Figure 1c,d depict the variation of the SE spectral signal peak height at the 1 and 2 kV primary beam voltages and are plotted as a function of sample atomic number. These results were taken from 10 different test specimens with multiple metal foils in the same holder. Gold foil was present in each sample holder set, serving as a control sample, so that variations caused by specimen holder exchange were suppressed; see Supplementary Sect. 2 for more details. In order to obtain the SE spectral signal height, the background signal taken at 0 eV pass energy is subtracted from each spectra.

Figure 1c,d indicate that the SE spectral signal height for both 1 and 2 kV primary beam voltages varies approximately linearly with increasing atomic number, with more deviation from the straight line fit occurring for 1 kV compared to 2 kV. These results point towards the possibility of SE energy spectroscopy providing the means to estimate the atomic number of different elemental metal regions. In principle, SE spectral height information can be superimposed on to the conventional SE detector image in order to map atomic number variations over the surface of the specimen. A control metal region can in principle be incorporated into the specimen holder to facilitate calibration. The reason why previous studies on SE emission were not able to find a simple similar dependence with sample atomic number is that they were mainly directed towards monitoring SE yield, a bulk signal that is formed from SEs emitted over a wide range of different emission angles and energies^17,18^. SE energy spectroscopy however, provides the opportunity of examining how material contrast varies as a function of emission energy, and this extra information has made it possible to find simple quantitative relationship with sample atomic number. Even where the SE energy spectra were obtained by dedicated UHV SE energy spectral instruments on a range of different metal samples in the past, no such simple relationship with sample atomic number was reported^37^. One possible explanation for this may come from the SE energy spectrum’s dependence on instrument operating parameters such as the primary beam current and specimen chamber vacuum level. The procedure of using a common control metal in the sample holder for all sample measurements used here, seems to be an effective way of suppressing uncertainties created by these kinds of parameters changing.

The linear trend shown in Fig. 1c,d is not precise enough to distinguish between materials that have close atomic numbers. This is illustrated by examining the spectral peak heights of Au and Pt. At a primary beam voltage of 1 kV, Pt has a greater spectral peak height than Au, while at 2 kV, its spectral height is lower than the one for Au. One way of enhancing the amount of material contrast contained in the SE energy spectral signal is to negatively bias the specimen and its surrounding electrode. This technique accelerates the SEs and increases their kinetic energies as they pass through the analyser. The technique was previously reported as a means of improving the analyser output signal-to-noise ratio, and making the SE peak narrower and more symmetric. It was used to enhance dopant contrast and surface/trapped charge contrast^45–48^. Figure 1e,f demonstrate how biasing the specimen and its surrounding electrode by − 10 V in this context is similarly capable of enhancing material contrast information at a primary beam voltage of 1 kV, causing the negatively biased Au spectral signal height to be greater than the one for Pt, while making the Pt spectral signal broader than the Au spectral signal, overcoming the inconsistent results of the unbiased case shown in Fig. 1a,c.

**Figure 2:**
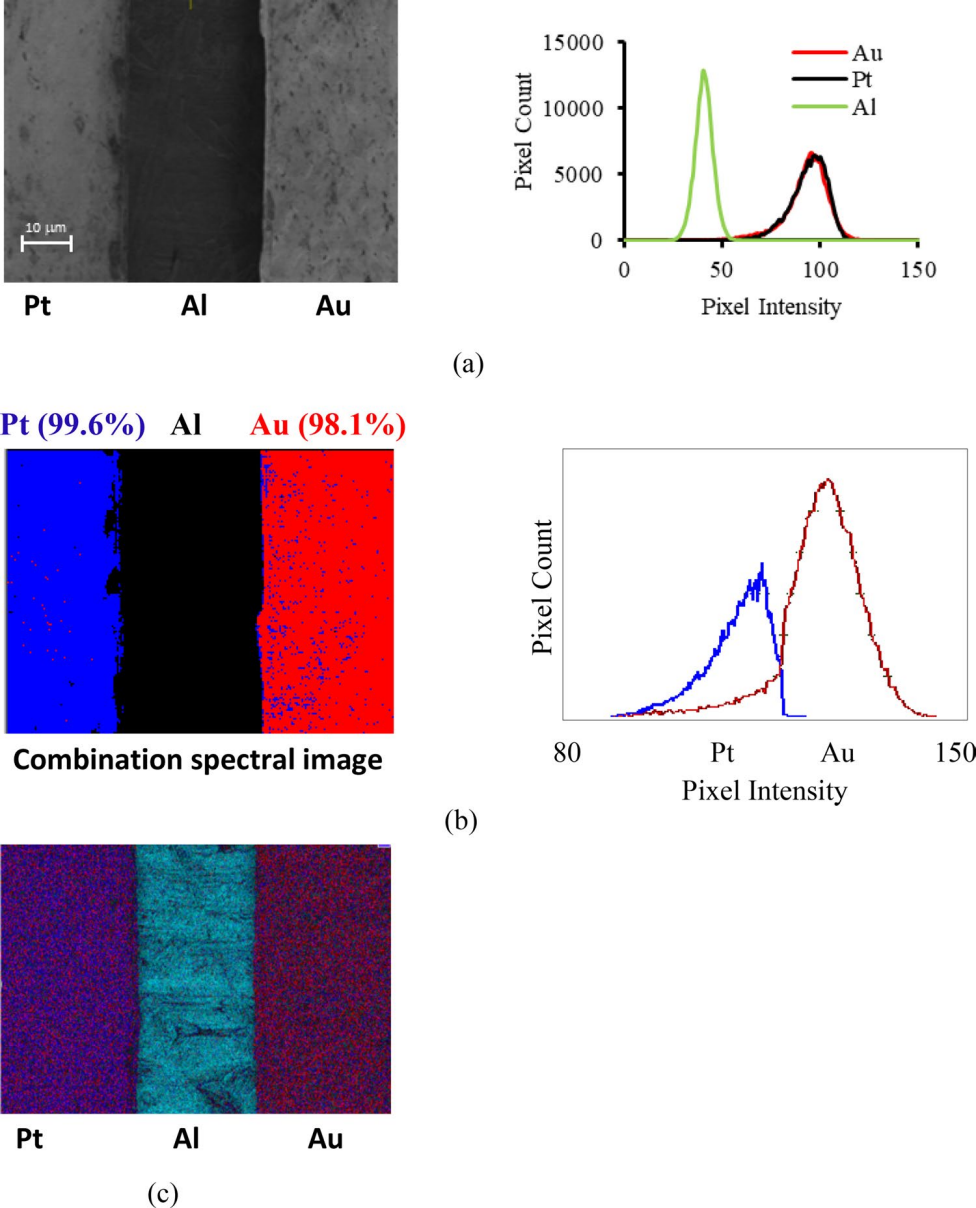
Quantitative material contrast mapping of Pt/Au foils on a Al base plate at a primary beam voltage of 1 kV (**a**) Conventional SE detector image/pixel intensity histogram. (**b**) Combination spectral image/pixel intensity histogram for k = 0.5. (**c**) EDX mapping at 2.5 kV.

The two material contrast spectral effects shown in Fig. 1e,f can be combined together in order to more accurately distinguish between Pt and Au regions on a sample, as demonstrated by the experimental results shown in Fig. 2. Material contrast mapping at a primary beam voltage of 1 kV is performed on a sample consisting of Au and Pt regions placed close together on an Al base plate. The SE spectral image *P*(i,j), was obtained from combining spectral peak height *h*(i,j), and standard deviation *σ*(i,j) information extracted from four separate images taken at the pass energies of 2.55 eV, 4.22 eV, 5.9 eV, and 7.57 eV, and translating it into an effective pixel intensity, P(i,j) = *h*(i,j)- *k*σ(i,j), where the constant *k* was varied in order to maximise material contrast.

Figure 2a shows a conventional SE detector image with its pixel intensity histogram of the Au–Al–Pt test specimen. As expected, the intensities of the Au and Pt regions in the image are indistinguishable, and their respective pixel intensity histograms lie on top of one another. Figure 2b shows similar results from the combined SE spectral technique, which is colour coded in terms of the combination function P(i,j) intensity. In this case, since there is clear separation between the pixel intensity Pt and Au histograms, a threshold intensity value separating blue and red intensity ranges is set at the mid-intersection point between the two histograms, resulting in near 100% material contrast separation: 99.6% (blue) for the Pt region and 98.1% (red) for the Au region. Figure 2c shows the corresponding EDX image, taken at a primary beam voltage of 2.5 kV. Although this value is considerably higher than the 1 kV primary beam voltage used for the SE spectral image, there is very poor material contrast in the EDX map, with almost no discernible visible difference between the Pt and Au regions of the sample. The experimental results shown in Fig. 2 demonstrate how tracking small changes in the − 10 V biased SE energy spectral signal shape provides the possibility of carrying out quantitative material mapping for LVSEM, and overcomes the present limitations of both conventional SE imaging and EDX attachments. At a primary beam voltage of 5 kV, EDX map is able to effectively distinguish between Au and Pt regions on the specimen and gives material contrast that is comparable to the combination energy spectral image, while in the SE detector image, the Au and Pt regions still have indistinguishable intensities. See Supplementary Fig. 3 for the 5 kV primary beam voltage images.

### SE spectral fine features and the valence band bulk density of states (DOS) distribution

**Figure 3:**
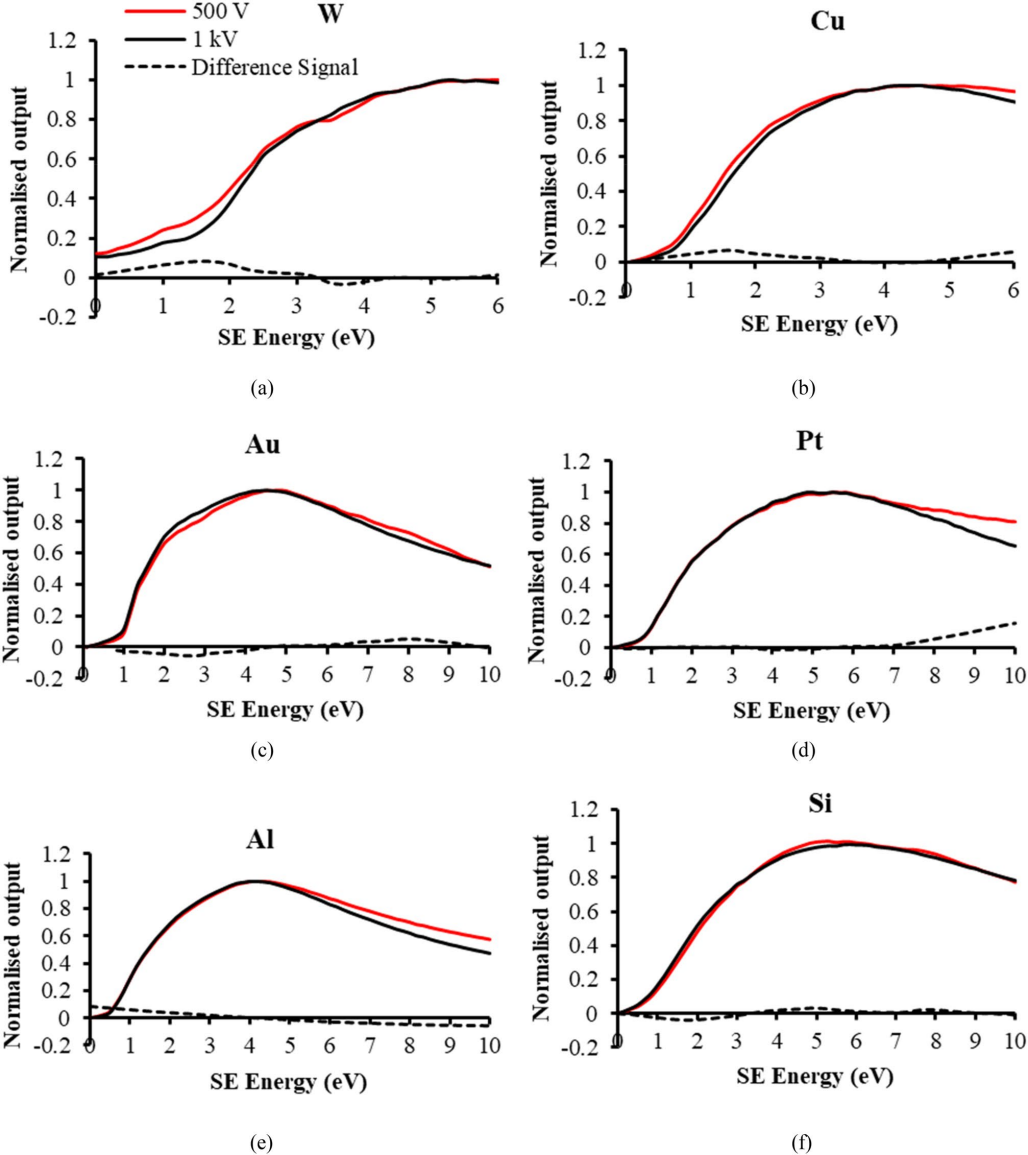
Normalised analyser spectral signals at the primary beam voltages of 1 and 0.5 kV and their difference signals for 6 test specimens: (**a**) W (**b**) Cu (**c**) Au (**d**) Pt (**e**) Al (**f**) intrinsic Si.

**Figure 4:**
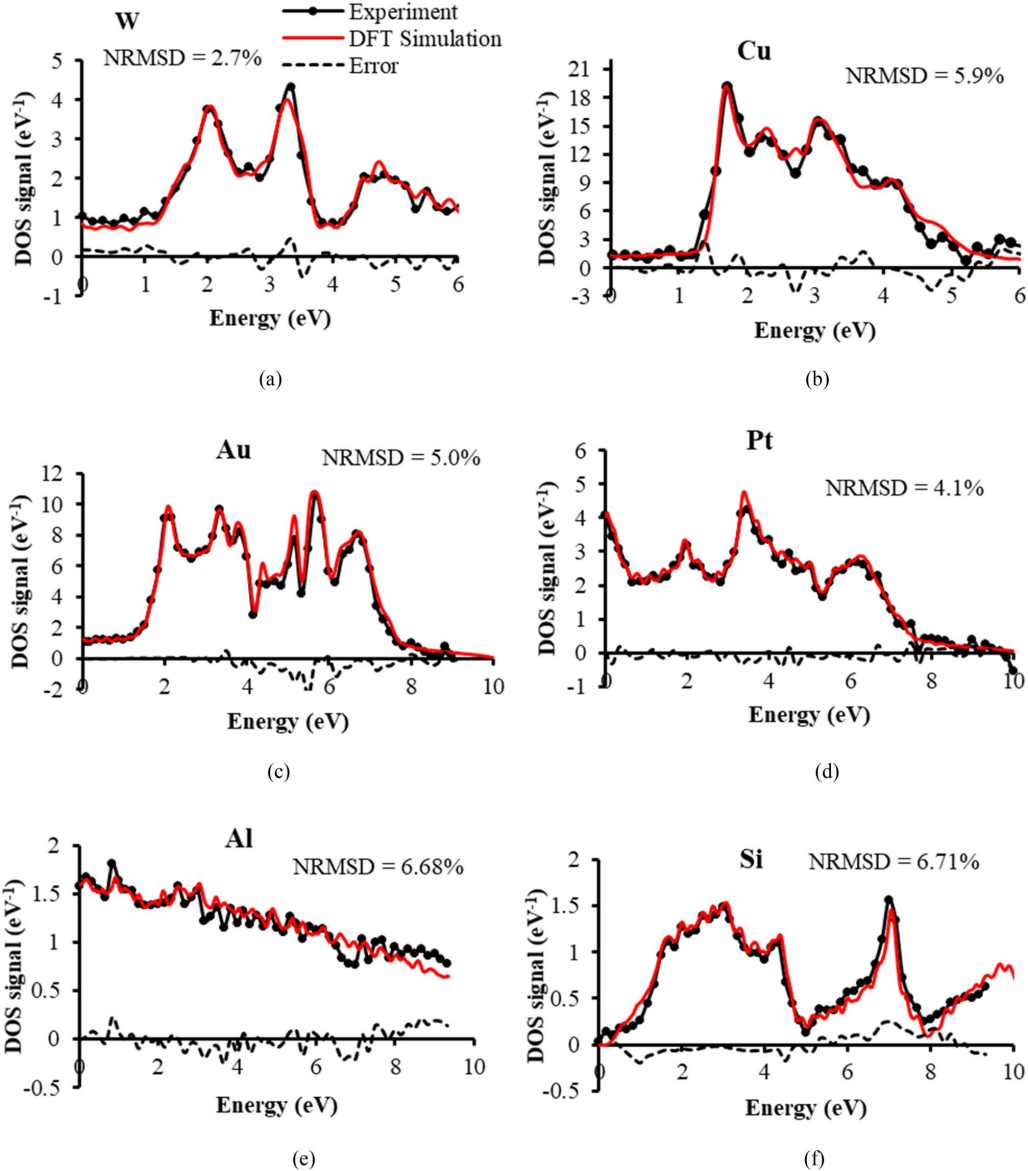
Comparison of SE spectral experimental DOS signals with theoretical DFT distributions and their error signals for 6 test specimens. The independent variable (“Energy”) is labelled as defined by DFT distributions, but experimentally corresponds to SE energy. The experimental SE spectral DOS signal is obtained by numerically differentiating the difference signals shown in Fig. 3. The NRSMD (normalised root mean square difference) values of the error signals are normalised to the experimental DOS signal peak value: (**a**) W (**b**) Cu (**c**) Au (**d**) Pt (**e**) Al (**f**) intrinsic Si.

An experimental valence band DOS spectral signal, *S*_*DOS*_*(E),* is obtained from the following procedure:

$${S}_{DOS}\left(E\right)=\frac{d}{dE}\left[{S}_{0.5kV}\left(E\right)-{S}_{1kV}\left(E\right)\right]=\frac{d{S}_{V}(E)}{dE}$$

where, *S*_*0.5 kV*_*(E)* is the 0.5 kV primary beam voltage normalised spectral signal, *S*_*1kV*_*(E)* is the 1 kV primary beam voltage normalised spectral signal, and *S*_*V*_*(E)* is the residual difference spectral signal between the two. The rationale for doing this comes from the observation that fine structure spectral features appear in *S*_*0.5 kV*_*(E)* when compared to *S*_*1kV*_*(E)*, as indicated by the experimental spectral signals of 6 different test specimens shown in Fig. 3a–f. The residual difference signals, *S*_*V*_*(E)*, differ from one another, suggesting that they are characteristic of the test material being examined. When the difference signal *S*_*V*_*(E)* is differentiated with respect to SE energy *E*, it closely matches the corresponding simulated bulk DFT distribution for each test sample, as shown in Fig. 4. The Fermi Energy is taken here to be 0 eV, and the bound occupied energy states normally depicted below the Fermi level are plotted to the right, in the direction of increasing SE energy. It is assumed here that the energy released in breaking valence band bonds is translated directly into an additional SE kinetic energy. The close match of experiment with theory suggests that this information comes mostly from within the specimen bulk region. These results also indicate that the background SE cascade interactions behind each of the *S*_*0.5 kV*_*(E)* and *S*_*1kV*_*(E)* spectral signals are approximately of the same kind and cancel out in the expression for the normalised residual difference spectral signal *S*_*V*_*(E)*, leaving information that comes directly from the density of states function *g(E)*. Differences in the SE yield or interaction volume between the 0.5 and 1 kV primary beam voltage spectral signals seem to be suppressed by normalising their respective spectral signals.

Changes in *ΔS*_*V*_*(E)* coming from the valence band density of states *g(E)*, assuming that they are all occupied, take the form $\Delta {S}_{V}(E)\approx g(E)\Delta E$, where *ΔE* is the energy analyser detection width, and this explains why the experimental density of states is given approximately by the gradient of the residual difference signal *S*_*V*_*(E)* for small *ΔE*:

$$g\left(E\right)\approx {S}_{DOS}\left(E\right)=\frac{\Delta {S}_{V}(E)}{\Delta E}\approx \frac{d{S}_{V}(E)}{dE}.$$

At the energy analyser resolution of 2.5%, the energy width *ΔE* (e.g. 0.025 eV at 1 eV pass energy, and 0.25 eV at 10 eV pass energy) is smaller than or comparable to the pass energy step size of 0.167 eV used for the 0–10 eV SE range, and the gradient distributions shown in Fig. 4 were therefore obtained by numerical differentiation. The high accuracy of match achieved between experimental DOS signals and the simulated DFT distributions is illustrated by their respective NRSMD values all being below 7%.

The experimental DOS spectral signals shown in Fig. 4 were obtained after investigating the effects of specimen acid pre-cleaning, beam induced contamination, and output spectral signal averaging. Strategies to minimise these effects were taken in order to lower the NRSMD to below 7% for each test sample. The results in Fig. 4 therefore represent optimal experimental DOS distributions for each test sample. In the case of W, there was no need for acid cleaning, however for other samples, acid pre-cleaning was required. Due to the relatively lower yield of the Al and Si samples, they required a higher degree of spectral signal averaging, 36 spectra for Al, and 48 spectra for Si, compared to the 12 to 15 spectral averaging used for W, Cu, Au and Pt test samples. 12 spectra were used for the W, Cu and Pt specimens, and 15 spectra were used for Au. It is interesting to note that after HF pre-cleaning in the case of the intrinsic Si sample, a conductive path to ground was required, which was achieved through the use of a carbon tape connecting the sample surface to the grounded surrounding holder, while for the uncleaned Si sample, no carbon tape conduction path was required.

**Figure 5:**
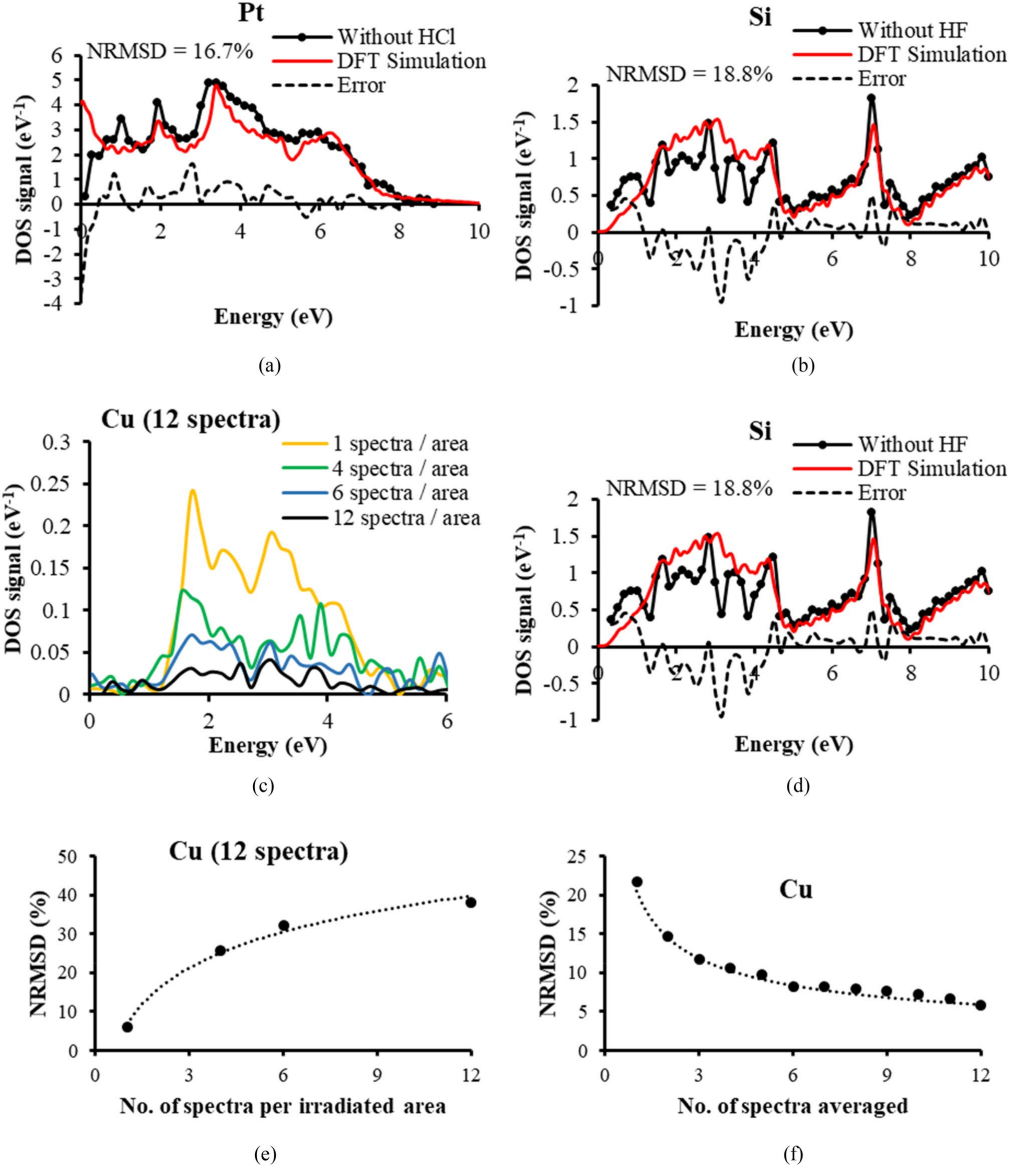
Effects of acid pre-cleaning, contamination, and spectral signal averaging on the accuracy of the experimental SE spectral DOS signals (**a**) Pt specimen without HCL pre-cleaning (**b**) Intrinsic Si without HF pre-cleaning (**c**) Variation in contamination on Cu by altering the number of irradiated areas for fixed number of 12 spectra (**d**) Match to theoretical DFT distribution on Cu for 12 spectra all from one irradiated area (**e**) NRMSD variation with number of spectra per irradiated area on Cu for 12 spectra. Dotted fitted curve takes the form, $NRMSD=13.3\%*\mathrm{ln}\left({N}_{area}\right)+6.6\%$ (f) NRMSD variation with the number of spectra averaged, dotted fitted curve takes the form: $NRMSD=\frac{20.4\%}{\sqrt{N}}.$

Examples of the error between experimental and theoretical DOS distributions for no pre-cleaning of the Pt and intrinsic Si samples are given in Fig. 5a,b respectively: the NRMSD increases to 16.7% for Pt (from 4.1%), and to 18.8% for intrinsic Si (from 6.71%). Corresponding results for the uncleaned Cu sample are given in Supplementary Fig. 4a, where its NRSMD was found to be 12.2%, up from 5.9% for the HCl cleaned sample. In the case of the Pt sample, it is interesting to note how significant errors occur for low SE energies (< 1 eV), causing the DOS spectral signal to fall as it approaches zero energy, when in fact it should rise. In the case of intrinsic Si, the errors caused by not applying HF pre-cleaning are also in the lower SE range, from 0 to just above 4 eV.

Improvements in the match between the experimental DOS spectral signals and their corresponding simulated DFT distributions caused by specimen acid pre-cleaning highlight an important point about the present SEM SEES technique. They indicate that while most the information of the experimental DOS spectral signals comes from the specimen bulk region, some of it also originates from the specimen surface, and that the surface contribution can be minimised by specimen surface pre-cleaning. The opposite situation appears to be true for the PES technique, where most its information comes from specimen surface, and precautions need to be undertaken in order to minimise bulk specimen effects^49^.

The influences of beam induced amorphous carbon contamination on the DOS spectral signals are given in Fig. 5c,d for the Cu test specimen. In this case, the number of spectra per irradiated area on the specimen was varied, while keeping the total number of spectra to be 12. The greater number of spectra per irradiated area therefore represents a higher level of beam induced contamination: the lowest level of contamination is produced by one irradiated area per spectrum, while the highest level of contamination comes from all 12 spectra being recorded from the same irradiated area. Figure 5c indicates that as the level of contamination increases, the overall height of the spectral signal falls. This is consistent with other investigations of contamination effects inside the SEM^46,59,60^. However, the form of the spectral signal also changes, as indicated in Fig. 5d where 12 spectra are recorded from a single irradiated area, and this increases the NRMSD to 38.2% (from 5.9%). The errors caused by increased levels of contamination appear throughout the 0 to 6 eV energy range, but seem to be highest in the lower SE range, from 0 to 4 eV.

Figure 5e provides more information about the way beam induced contamination imposes errors. The NRMSD seems to vary logarithmically as a function of increasing levels of contamination (number of spectra per irradiated area). This is in agreement with prior investigations that monitored changes in shape of the SE energy spectral signal as levels of contamination were increased on doped Si samples^46^. It indicates that there is a fast contamination layer rate of growth initially, but it then subsequently slows down. Figure 5f quantifies the effects of shot noise by monitoring how the NRMSD changes as a function of the number of spectra used in the final averaging procedure. These results are consistent with prior experience of the role of shot noise in the SEM, both for SE imaging and SE spectral acquisition, where shot noise signal-to-noise ratio (SNR) have been observed to follow 1/√N Poisson/Normal distribution statistics^46,61,62^. The corresponding shot noise effects for Al and Si are presented in Supplementary Fig. 4b,c respectively, and although they required much higher levels of averaging, they also followed the same kind of shot noise statistics. Knowledge of how beam induced contamination and shot noise errors change the NRMSD in a quantitative way, help in the selection of experimental operating parameters in order to achieve a given DOS signal accuracy.

**Figure 6:**
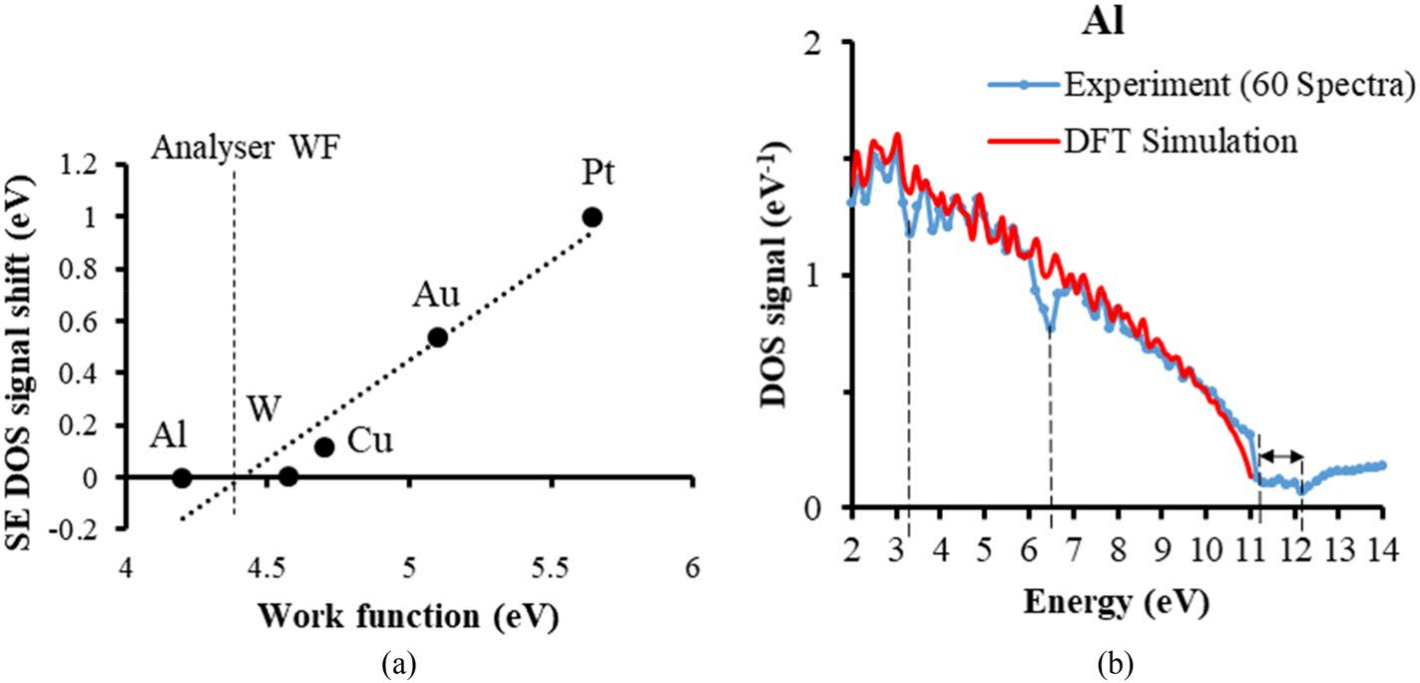
(**a**) Energy shift variation with sample work function. The analyser work function value of 4.4 eV is found from the fitted line intercept with the work function axis. The work function values for each metal element were obtained from Michaelson^80^, and Hölzl and Schulte^81^. The quoted accuracy for the work function values are less than ± 0.1 eV. (**b**) Energy loss information superimposed upon the experimental DOS signal, at the energies, 3.3, 6.5 and 11–12 eV.

While matching the experimental DOS spectral signals to their theoretical DFT counterparts, opportunities for acquiring additional information were found. There was for instance, an overall consistent energy shift obtained for some test metal samples. The cause for this energy shift most likely comes from there being an analyser work function created by the different metal junctions in the conductive path between the specimen and the analyser deflection plates. A very similar effect has been observed in the case of PES measurements^63^, where the size of the energy shift has been equated to be the difference between the specimen work function and analyser work function. Figure 6a shows a plot of the energy shift obtained here as a function of the specimen work function for the 5 metals used. The fitted linear variation of the energy shift value to the specimen work function value makes it possible to estimate the analyser work function value, found here to be 4.4 eV from Fig. 6. This information may be helpful in understanding the energy shift obtained for other specimens.

Another extra piece of information obtained while matching the experimental DOS signals to their corresponding theoretical DFT distributions was the consistent appearance of some energy loss features in the DOS signal, see for instance the Al DOS signal shown in Fig. 6b. In order to reduce the effect of shot noise, the number of spectra averaging was increased to 60 (from 36), and the energy range was extended to 14 eV. There appear to be two clearly identifiable energy loss features in the 0 to 14 eV range, one which has a minimum located at 6.5 eV, and another having a minimum between 11 to 12 eV. In the past small discontinuities created by surface/volume plasmon interactions in the SE energy spectrum of Al were found by experiment and predicted by Monte Carlo simulations to be around 5.5 eV (surface plasmons) and 10 to 10.5 eV (volume plasmons)^38,39,41,64–66^. However, the present results are more accurately compared to those presented by Pillion et al.^67^, where the SE energy spectrum was differentiated and the positions of the energy loss minima were found to lie at 6.4 eV (surface plasmons) and 11.2 eV (volume plasmons). In this case, for the volume plasmon energy loss feature, there is a sharp dip down that occurs at 11.2 eV, but it then seems to extend to12 eV before rising again. There also seems to be a smaller energy loss feature present at 3.3 eV, but this requires further investigation in order to better distinguish it from shot noise. Overall, the present results are consistent with SE energy surface/volume plasmon features reported previously, while at the same time illustrating how energy loss information is superimposed upon the SE spectral experimental valence band DOS signals.

The technique presented here has several natural areas for future development. One such area is to investigate the possibility of developing angle-resolved SEES (ARSEES) in the SEM, so that it can directly acquire band-structure information in a manner similar to ARPES. This would require modifying the present wide-angle toroidal energy analyser attachment design so that it captures the azimuthal angular distribution of SEs, and decouple the present analyser attachment from the specimen stage, enabling the specimen to be moved, rotated and tilted. Another obvious line of development is to implement SE energy spectroscopy into in-lens LVSEM columns. Several electron energy analyser design proposals to do this have already been made and some of them have already obtained proof-of-concept spectra^55–58^. More ways of reducing beam induced contamination need to be investigated within the SEM specimen chamber, such as local heating of the specimen. This will allow the 2D mapping of experimental valence band DOS directly, without the need to irradiate similar multiple areas on the specimen for the purpose of reducing contamination and shot noise effects. There is also the wider subject of capturing SE loss energy loss information to acquire surface/volume plasmon and conduction band density of states of information. This can be done by increasing the present SE energy detection range, beyond the present 10 eV limit for DOS measurements, and using a smaller exit aperture to increase the analyser’s relative energy resolution.

## Conclusion

This paper has demonstrated how secondary electron energy spectroscopy (SEES) performed inside a scanning electron microscope (SEM) can be used to map sample atomic number and acquire bulk valence band density of states information, and how it has the potential to become a quantitative material science companion tool for LVSEM. It also points towards a new line of development for Scanning Auger Microscope (SAM) instruments. A relatively high level of accuracy has been achieved between experimentally derived SE spectral DOS signals and theoretical valence band DOS data for six different test samples, where the normalised root mean square deviation (NRMSD) was found to be below 7%. Such high levels of agreement between experimental and theoretical bulk valence band DOS data do not appear to have been reported before.

## Methods

### Instrumentation and test samples

The electron energy analyser SEM attachment design used here is a wide-angle band-pass toroidal electric sector that has second-order focusing properties^29^. In the current configuration of the analyser, the entrance polar angular spread is ± 8°, the entrance azimuthal angular spread is 100°, and the exit aperture size is 0.5 mm, giving a relative energy resolution of approximately 2.5% with an entrance solid angle capture of 0.687 sr, around 11% transport efficiency of 2π sr hemispherical emission^46^. The inner deflection plate of the analyser is grounded, and the analyser pass energy (E_P_) to outer plate voltage (V_d_) ratio is ${E}_{P}={V}_{d}/0.6$. More about how the analyser attachment is installed into the SEM and the way it is operated through LabVIEW software is given in Supplementary Sect. 1.

All SE spectroscopy experiments were carried out in an FEI Quanta 200 3D FIB-SEM using its tungsten filament electron source. The beam current used was 50 pA for both 500 V and 1 kV beams. The EDX experiments were performed in a Hitachi Regulus SU8230 FE-SEM with an Oxford Instruments Ultim Extreme 100 mm^2^ windowless EDX. The beam conditions used in these experiments were chosen to be as close to the SE spectroscopy experiments for accurate comparison.

The metallic foil samples, including W (99.95%, 0.01 mm), Cu (99.9%, 1.0 mm), Au (99.9%, 0.01 mm), Pt (99.95%, 0.01 mm) and Al (99.999%, 1.0 mm), were cut into 1 cm × 1 cm flat pieces and immersed in a 10% HCl solution for extended periods (~ 30 min) to remove possible presence of surface oxides. For 1.0 mm thick foils, brief ultrasonication while submerging the foil sample inside the HCl solution was also carried out using an ultrasonic cleaner, ultrasonic frequency of 40 kHz. The foils were subsequently rinsed in DI water and isopropyl alcohol and blown dry using a N_2_ gun.

The intrinsic, < 100 > silicon wafers (500 μm thickness, 10–100 Ω·cm resistivity) were diced into 1 cm × 1 cm pieces. For a typical HF treatment procedure, the wafer piece was immersed into a buffered oxide etchant (BOE) 6:1 solution for 2 min. It was subsequently rinsed with DI water several times and blown dry with a N_2_ gun. The wafer sample was then immediately loaded into a SEM chamber for pump-down and measurement minimizing air exposure.

### SE energy spectral signal acquisition

For the DOS experiments, the SE energy spectrum was acquired while scanning areas of 5 by 5 microns on the specimen surface. In order to reduce the effect of primary beam induced contamination on the specimen’s surface, one spectrum was obtained from a single area of scan, and the primary beam was then moved to a fresh area before recording another spectrum. Multiple spectra were therefore acquired from multiple irradiated 5 by 5 micron areas on the specimen. For the purposes of spectral signal averaging, up to 15 spectra were obtained from a single test specimen for the W, Cu, Au and Pt test specimens. Due to the lower yields of the Al and intrinsic Si samples, between 36 to 48 spectra were recorded. The overall SE energy scan rate per spectrum was also varied in order to minimise contamination effects, and it typically varied from 0.0625 to 0.25 s per deflection plate voltage/pass energy step, and the total acquisition time varied between 23 and 45 s per spectra. This spectral acquisition time included LabVIEW software dead times during which the output signal was not recorded. The total SE energy spectral time was varied according to the experiment being carried out: for obtaining experimental valence band bulk DOS, the spectra taken at 1 kV and 0.5 kV had spectral acquisition times up to 23 s for 61 points per spectrum over a 0 to 10 eV energy range, while for tracking the SE spectral peak height, a spectral acquisition time of around 20 s for 17 points over a 0 to 20 V energy range was used.

The analyzer system was operated in spectroscopic image mode for the close atomic number Au/Pt mapping experiments. A series of images were acquired, with each image being recorded for a different fixed deflection voltage/pass energy. XY scan control signals were applied via LabVIEW software, which was also used to simultaneously monitor the analyser detector output. This was done with a frame rate of 6 s/ frame, an image resolution of 500 × 242 pixels, and a primary beam current of 130 pA.

### The DFT simulation procedure

First-principles calculations were carried out within the framework of Hohenberg–Kohn–Sham density functional theory (DFT)^68,69^ using the generalized gradient approximation (GGA) in the parameterization of Perdew–Burke–Ernzerhof (PBE) format exchange–correlation functional^70^, as implemented in the Vienna ab initio Simulation Package (VASP)^71–74^. The projector-augmented wave (PAW) pseudopotentials were selected to describe the interaction between electrons and ions^75,76^. In all calculations, the kinetic energy cut-off for the plane wave basis was set to 600 eV and the criteria of electronic convergence was set at $1.0\times {10}^{-8}$ eV, which ensured the convergence of total electronic-ground-state energy. A very dense $23\times 23\times 23$ k-point mesh was sampled by the Monkhorst–Pack scheme for integration over the whole Brillouin zone. The experimental lattice constants were adopted in the structural relaxation, i.e., Al (#225, fcc, a = 4.050 Å), Si (#227, dia, a = 5.430 Å), Cu (#225, fcc, a = 3.615 Å), W (#229, bcc, a = 3.165 Å), Pt (#225, fcc, a = 3.924 Å), and Au (#225, fcc, a = 4.078 Å)^77,78^, where # indicates the space group number for each bulk crystal, fcc/bcc stands for face/body-centered cubic, dia refers to diamond cubic. The atomic positions were fully optimized until the Hellmann–Feynman force on each atom was less than 0.005 eV/Å.

In order to accurately calculate the density of states (DOS) of various elementary substances, which describes the number of states that are to be occupied by electrons at each level of energy, the corresponding electronic configurations were chosen: Al (s^2^p^1^, Z = 13), Si (s^2^p^2^, Z = 14), Cu (d^10^p^1^, Z = 29), W(p^6^d^6^s^2^ Z = 74), Pt (d^9^s^1^ Z = 78), and Au (d^10^s^1^ Z = 79), where Z is the atomic number. Spin–orbit coupling (SOC) contributions were included in all self-consistent calculations of DOS to account for the non-collinear spin polarization in each orbital. The Fermi–Dirac distribution^79^ was used as the smearing method to plot the DOS. We have also considered different smearing methods but the overall shape of DOS remained untouched. The width of the smearing was set to 0.05 eV.

## Supplementary Information

Supplementary Information
