# Author Correction: Quantitative material analysis using secondary electron energy spectromicroscopy

**DOI:** 10.1038/s41598-021-87188-w

**Published:** 2021-04-01

**Authors:** W. Han, M. Zheng, A. Banerjee, Y. Z. Luo, L. Shen, A. Khursheed

**Affiliations:** 1 Department of Electrical and Computer Engineering, National University of Singapore, 4 Engineering Drive 3, Singapore, 117583 Singapore; 2 Physics Department, Bidhan Chandra College, Kazi Nazrul University, Asansol, West Bengal 713303 India; 3 Department of Mechanical Engineering, National University of Singapore, 9 Engineering Drive 1, Singapore, 117575 Singapore

Correction to: *Scientific Reports* https://doi.org/10.1038/s41598-020-78973-0, published online 17 December 2020

This Article contains an error in Figure 5, where Figure 5d is a duplication of Figure 5b. The correct Figure 5 appears below as Figure 1.

**Figure 1:**
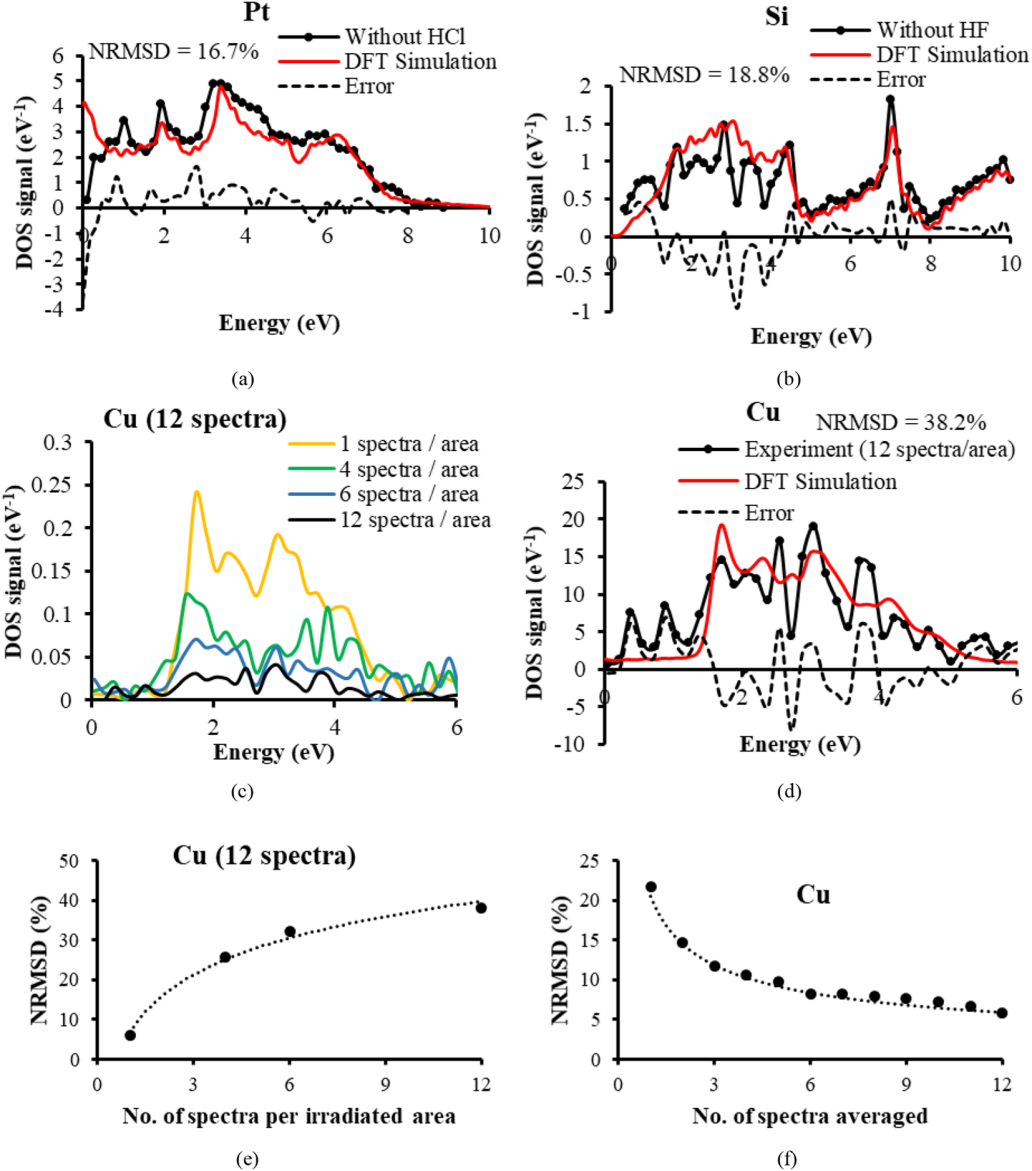
A correct version of the original Figure 5.

