# Supplementary material for: Quantitative material analysis using secondary electron energy spectromicroscopy
Source: Sci Rep. 2020 Dec 17;10:22144. doi: 10.1038/s41598-020-78973-0 (PMC7746715; doi:10.1038/s41598-020-78973-0)
Supplement: Supplementary file 1 — Supplementary Information [file 41598_2020_78973_MOESM1_ESM.docx]

**Quantitative material analysis using secondary electron energy spectromicroscopy**

W. Han^1^, M. Zheng^1^, A. Banerjee^2^, Y.Z. Luo^3^, L. Shen^3^, and A. Khursheed^1^*.

^1^Department of Electrical and Computer Engineering, National University of Singapore, 4 Engineering Drive 3, Singapore, 117583, Singapore.

^2^Physics Department, Bidhan Chandra College, Kazi Nazrul University, Asansol, West Bengal, India 713303.

^3^Department of Mechanical Engineering, National University of Singapore, 9 Engineering Drive 1, Singapore 117575.

*Corresponding Author. Email Address: eleka@nus.edu.sg

## Supplementary Section 1: The wide-angle toroidal analyser attachment

Supplementary Fig. 1a represents a schematic diagram of how the wide-angle toroidal energy spectrometer attachment is installed and operated inside a SEM specimen chamber. The attachment is mounted on the SEM stage, and aligned to the primary beam axis in-situ by using the SE detector image. The height of the stage is also adjusted such that the working distance to the sample surface is 15.5 mm. The deflection voltage was generated using an Agilent E3631A Power supply and the signal electrons are measured using a scintillator-PMT detector (Hamamatsu H10722-01) coupled to an NI USB-6356 DAQ. While each fresh scan area is manually selected through the SEM’s software, the deflection voltage and signal acquisition was automated using a custom LabVIEW program.

Since the SE energy spectrum is reconstructed by varying the analyzer pass energy, the accuracy of the captured SE energy spectrum naturally depends on how rapidly the spectral signal varies in comparison to the analyzer detection energy (width) *ΔE*. Energy dispersion at the analyzer output focal plane varies linearly with the pass energy, *E_P_*, so that the detected energy width is given by$\Delta E=k E_{P}$, where *k* is a constant. The analyzer signal is therefore inherently different in form to the SE emission energy distribution. At any given SE energy, the analyzer signal is integrated over a finite bandwidth *ΔE* around the pass energy *E_P_*, and since $\Delta E$ increases linearly with$E_{P}$, the analyzer spectral signal $S(E)$ expressed in terms of an SE emission distribution $N(E)$ is given approximately by, $S\left( E \right)\approx N\left( E \right).\Delta E = k.N\left( E \right).E$. This formula assumes that $\Delta E$ is relatively small. Supplementary Figure 1b shows both the normalized theoretical SE emission distributions for gold and silicon and their corresponding theoretical SE analyzer signals, where the Chung and Everhart (1974) distribution is used for gold, and the Henke *et al.* (1979) distribution is used for silicon. The work function (*W*) for gold is taken to be 5.1 eV and the electron affinity χ for silicon is 4.05 eV. The theoretical analyzer signals are much broader than the SE emission distribution, caused by the fact that the analyzer signal is proportional to$N(E).E$*,* not$N(E)$*.* For metals, the SE peak position for *N(E)* should occur at *W*/3, while for *S(E)*, it lies at *W*.


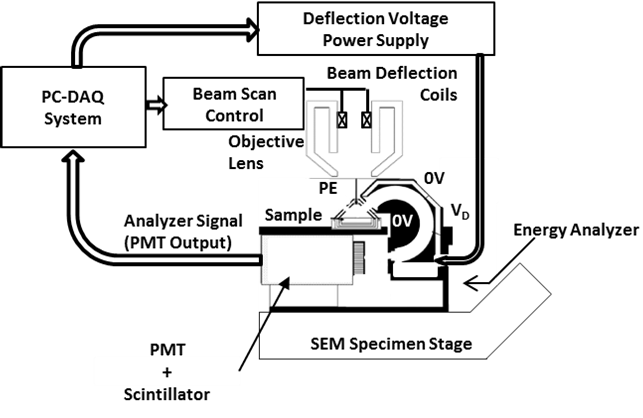


(a)

(b)

Supplementary Figure 1 (a) Analyzer system schematics (b) Analyzer signals compared to Chung-Everhart distribution

## Supplementary Section 2: The specimen holder layout

Metal foil samples were cut into 6 x 6 mm right-angle triangles and aligned on the sample holder, as illustrated in Supplementary Figure 2. This was to allow multiple foils to enter the same analyser field of view at the same time, making it possible to calibrate all foils to a single metal foil type. This is necessary, since vacuum conditions and the primary beam current vary between specimen exchanges. Gold foil was chosen as the control metal foil in these experiments due to its relatively inert nature. An aluminium cover was added to reduce tertiary electron generation due to scattering.


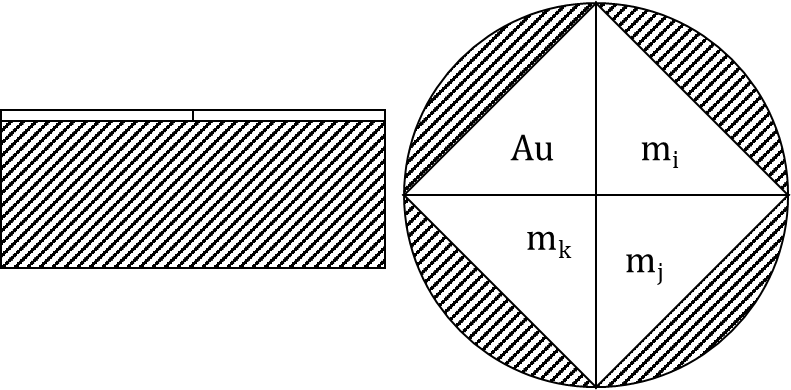


(a)


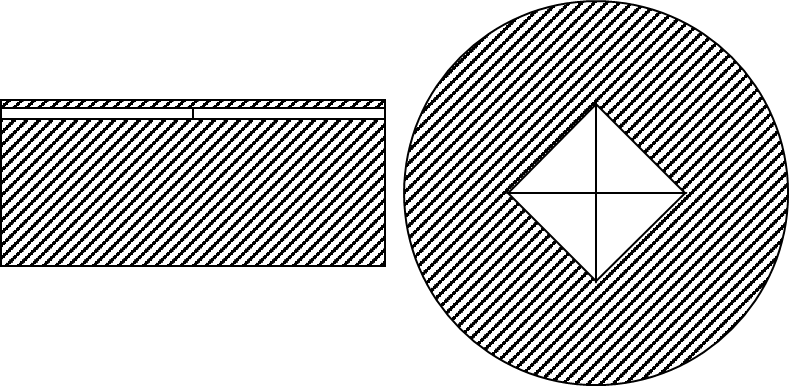


(b)

Supplementary Figure 2 Layout of metal foils in sample holder. (a) Without aluminium cover (b) With aluminium cover

## Supplementary Section 3: Material Mapping at 5 kV

Figure 3 shows quantitative material contrast mapping of the Au-Al-Pt test sample at a primary beam voltage of 5 kV. In this case, the EDX image is effective in being able to differentiate between the Au and Pt regions, while the SE detector image is still unable to distinguish between them. The combination spectral image has near 100% material contrast between the Au and Pt regions, and has the added advantage of also displaying surface topography.

**Pt Al Au**


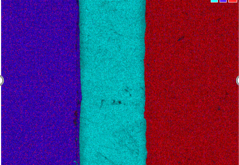


**Pt Al Au**

(a)

(b)

(c)


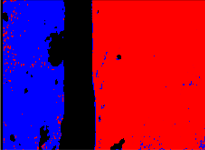


**Pt (97.9%) Al Au (99%)**

**Combination spectral image**


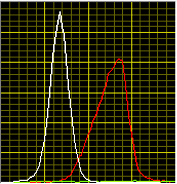


Pixel Count

80 Pt Au 130

Pixel Intensity

Supplementary Figure 3 Quantitative material contrast mapping of Pt/Au foils on an Al base plate at a primary beam voltage of 5 kV

(a) Conventional SE detector image/pixel intensity histogram.

(b) Combination spectral image/pixel intensity histogram for k = 0.5.

(c) EDX mapping at 5 kV.

## Supplementary Section 4: Error observations on the experimental DOS distributions

Figure 4a shows the increase in error between the experimental DOS signal and its corresponding DFT distribution when no HCl plre-cleaning treatment was applied to the Cu sample. The NRMSD error grows to 12.2%, uo from 5.9% when HCl cleaning is used. Figures 4b records shot noise characteristics for the Al sample, while Figure 4c records it for the Si sample. In both cases, the NRMSD error follows expected shot noise 1/√N statistics.

(a)

(b) (c)

Supplementary Figure 4 Effects of acid pre-cleaning and spectral signal averaging on the accuracy of the experimental SE spectral DOS signals for other elements

(a) Cu specimen without HCL pre-cleaning

(b) NRMSD variation with the number of spectra averaged for Al, with fitted curve (dotted) $NRMSD=\frac{66.2\%}{\sqrt{N}}$

(c) NRMSD variation with the number of spectra averaged, with fitted curve (dotted) $NRMSD=\frac{41.8\%}{\sqrt{N}}$
